# The Public Health Insurance Coverage of Novel Targeted Anticancer Medicines in China—In Favor of Whom? A Retrospective Analysis of the Insurance Claim Data

**DOI:** 10.3389/fphar.2021.778940

**Published:** 2021-12-21

**Authors:** Mingshuang Li, Yifan Diao, Jianchun Ye, Jing Sun, Yu Jiang

**Affiliations:** ^1^ School of Population Medicine and Public Health, Chinese Academy of Medical Sciences and Peking Union Medical College, Beijing, China; ^2^ School of Health Policy and Management, Chinese Academy of Medical Sciences and Peking Union Medical College, Beijing, China; ^3^ Healthcare Security Administration of Fujian Province, Fuzhou, China

**Keywords:** targeted anticancer medicines, basic health insurance, benefits package, real-world, interrupted time series, multivariate linear regression, gefitinib, icotinib

## Abstract

**Objectives:** This study took Fuzhou city as a case, described how the public health insurance coverage policy in 2016 of novel anti-lung cancer medicines benefited patients, and who benefited the most from the policy in China.

**Methods:** This was a retrospective study based on health insurance claim data with a longitudinal analysis of the level and trend changes of the monthly number of patients to initiate treatment with the novel targeted anti-lung cancer medicines gefitinib and icotinib before and after health insurance coverage. The study also conducted a multivariate linear regression analysis to predict the potential determinants of the share of patient out-of-pocket (OOP) expenditure for lung cancer treatment with the study medicines.

**Results:** The monthly number of the insured patients in Fuzhou who initiated the treatment with the studied novel targeted anti-lung cancer medication abruptly increased by 26 in the month of the health insurance coverage (95% CI: 14–37, p < 0.01) and kept at an increasing level afterward (p < 0.01). By controlling the other factors, the shares of OOP expenditure for lung cancer treatment of the patients who were formal employee program enrollees not entitled to government-funded supplementary health insurance coverage and resident program enrollees were 18.3% (95% CI: 14.1–22.6) and 26.7% (95% CI: 21.0–32.4) higher than that of the patients who were formal employee program enrollees with government-funded supplementary health insurance coverage.

**Conclusion:** The public health insurance coverage of novel anti-lung cancer medicines benefited patients generally. To enable that patients benefit from this policy more equally and thoroughly, in order to achieve the policy goal of not to leave anyone behind, it is necessary to strengthen the benefits package of the resident program and to optimize the current financing mechanism of the public health insurance system.

## Introduction

Cancer has been the leading cause of death since 2010 in China (Chen et al., 2012; Zeng et al., 2012; Chen et al., 2016). The new cases of and deaths from cancer in China accounted for 23.7% and 30.2% of the total global numbers respectively in 2018 (Zheng et al., 2019). China’s cancer survival rate is far below the average of developed countries (Allemmani et al., 2018; Zeng et al., 2018; Sigel et al., 2020). Apart from early diagnosis and detection, the rapid application of targeted anticancer medicines and technologies with outstanding clinical efficacy has made an essential contribution to the improvement of the cancer survival rate (Ma et al., 2019). Taking lung cancer as an example, which has the highest morbidity and mortality in China, more than 80% of lung cancer patients are with non-small cell lung cancer (NSCLC), about 70% of NSCLC patients are in advanced stages at the time of diagnosis and have lost the opportunity for surgical treatment. New targeted medicines broke through the bottleneck of traditional chemotherapies (Shi et al., 2016). In addition to the delayed market approval, affordability is another key barrier to the timely uptake of the new targeted anticancer medicines. Most of the targeted anticancer medicines are expensive and not covered by the public basic health insurance before 2016 in China and was not affordable to most of the families (Wang, 2016; IQVIA Institute, 2018). To enable the Chinese cancer patients to get timely access to affordable novel targeted medication, the Chinese government developed a series of policies, including having those with outstanding clinical efficacy covered by the basic health insurance program (National Health and Family Planning Commission, 2016; Fang et al., 2017; Ministry of Human Resources and Social Security, 2017; Economic Information Daily, 2018; Health News, 2018; National Healthcare Security Administration, 2018).

Although the prices of the health insurance newly covered novel targeted anticancer medicines were reduced by at least 50% on average during the last four rounds of national price negotiations in China (National Health and Family Planning Commission, 2016; Ministry of Human Resources and Social Security, 2017, 2018, 2019), the financial burden of some patients may still be too high, as the initial market prices of these targeted anticancer medicines were very high (Diao et al., 2019). Patients who remain susceptible to the financial burden of cancer treatment still exists even in high-income countries (Ezeife et al., 2018). Higher out-of-pocket (OOP) costs were associated with increased rates of abandonment of insurance-covered novel anticancer agents (Doshi et al., 2018). The government has been trying to strengthen the financial safety guard of cancer patients and not to leave anyone behind (Central People’s Government of the People’s Republic of China, 2020). There is a need to generate empirical evidence about who benefited the most from the policy, who were likely to be left behind, and to what extent the patients’ benefit of the policy was associated with their respective benefits packages of the basic health insurance system. However, minimal empirical evidence is available (Chen et al., 2018; Xian et al., 2019; Diao et al., 2021).

Considering that China’s basic medical insurance is currently pooled and operated at the municipal level, this study took Fuzhou, the capital city of Fujian province with a median development level in southern China, as a case. The study focused on novel targeted therapies for advanced or metastatic epidermal growth factor receptor (EGFR) mutation-positive NSCLC covered by the basic health insurance program of Fujian province in December 2016, measured the changes of demographic and sociological distributions of the insured patients treated with the novel targeted anti-lung cancer medicines before and after the public health insurance coverage, and analyzed the determinants of patient financial burden.

## Materials and Methods

### Studied Medicines, Population, Setting, and Data Source

This study targeted gefitinib and icotinib, the first two targeted anti-lung cancer therapies covered by the basic health insurance program of Fujian province in November 2016. These two novel targeted medicines were covered as the first-line therapies for the indication of EGFR mutation-positive advanced or metastatic NSCLC (National Health Commission of the People’s Republic of China). This study focused on advanced or metastatic EGFR mutation-positive NSCLC patients enrolled in Fuzhou basic health insurance programs. Data of the targeted patients from January 2016 to December 2018 were extracted from the insurance claim database of the basic health insurance program of Fuzhou. The extracted data included patients’ personal information and their medical records of all lung cancer-related outpatient and inpatient care during the observation time.

### Study Design

This was a retrospective analysis based on the health insurance claim data. Considering that the actual implementation of the insurance coverage policy might lag behind the insurance coverage time (November 2016), we took January 2017 as the time point of policy intervention. The study period was divided into pre-intervention (January 2016–December 2016) and after-intervention (January 2017–December 2018) periods.

### Measurement

We measured the monthly number of patients who initiated the targeted treatment. Patient affordability was calculated as the share of OOP expenditure for the targeted treatment of lung cancer.

### Statistical Analysis

We used SPSS 25.0 software to perform the following tasks: 1) descriptive analysis of the demographic and socialogical distributions of the patients who initiated the treatment with the two novel targeted anti-lung cancer medicines before and after the public health insurance coverage. We performed chi-square tests to compare the differences of the demographic and sociological distributions of patients who initiated the treatment with the medicines of interest between 2017 and 2018, the first 2 years of the implementation of the public health insurance coverage policy. We also compared the differences in demographic and sociological distributions between patients who were treated with gefitinib and patients who were treated with icotinib during the study period of 2016–2018. We performed Bonferroni corrections for the pairwise comparisons; 2) segmented linear regression analysis of the level and trend changes of the monthly number of patients who initiated treatment with the medicines of interest before and after the public health insurance coverage; 3) multivariate linear regression analysis to predict the determinants of the proportionate OOP expenditure for lung cancer-related treatment with the medicines of interest. We adopted “enter” method to fit the regression model. The statistical significance level was set at p < 0.05.

## Results

### Demographic and Sociological Distributions of Patients Who Initiated Treatment With the Medicines of Interest Before and After Health Insurance Coverage

As presented in Table 1, a total of 883 basic health insurance beneficiaries adopted the medicines of interest from January 2016 to December 2018 in Fuzhou city. Before health insurance coverage, only 10 patients initiated treatment with the medicines of interest. This number increased to 873 afterward, composed of 396 in 2017 and 477 in 2018. The number of patients who initiated treatment with the medicines of interest in all demographic and sociological subgroups increased in 2017. There were statistically significant differences in the distributions of type of health insurance coverage, gender, employment status, and local or non-local medication between 2017 and 2018 (p = 0.03, p = 0.02, p = 0.03, p < 0.01, respectively). During 2016–2018, 608 patients adopted gefitinib and 275 adopted icotinib. There were statistically significant differences in the distributions of gender, local and non-local medication, and employment status between patients who adopted gefitinib and patients who adopted icotinib (p = 0.03, p < 0.01, p < 0.01, respectively).

**TABLE 1:** Demographic and sociological distributions of patients who initiated treatment with the medicines of interest before and after the public health insurance coverage (2016–2018).

**Demographic and sociological characteristics of patients included in the study**	**No. of patients *n* (%)**	**No. of patients who initiated treatment with medicines of interest before the public health insurance coverage**	**No. of patients who initiated treatment with medicines of interest after the public health insurance coverage**		**Test of the differences of distributions of patients who initiated treatment with medicines of interest between 2017 and 2018**		**No. of patients who initiated treatment with gefitinib (2016–2018) *n* (%)**	**No. of patients who initiated treatment with icotinib (2016–2018) *n* (%)**	**Test of the difference of distributions between patients who initiated treatment with gefitinib and patients who initiated treatment with icotinib (2016–2018)**	
		**2016 *n* (%)**	**2017 *n* (%)**	**2018 *n* (%)**	***χ* ^*2*^**	***p***			***χ* ^*2*^**	***p***
Type of health insurance coverage										
Formal employee program enrollee entitled with government-funded supplementary health insurance coverage	73 (8.3)	0 (0.0)	38 (9.6)	35 (7.3)	7.02	**0.03**	51 (8.4)	22 (8.0)	2.73	0.26
Other formal employee health insurance program enrollee	286 (32.6)	2 (20.0)	143 (36.1)	141 (29.6)			207 (34.0)	79 (28.7)		
Resident program enrollee	524 (59.3)	8 (80.0)		301 (63.1)			350 (57.6)	174 (63.3)		
Gender										
Male	395 (44.7)	6 (60.0)	159 (40.2)	230 (48.2)	5.70	**0.02**	257 (42.3)	138 (50.0)	4.80	**0.03**
Female	488 (55.3)	4 (40.0)	237 (59.8)	247 (51.8)			351 (57.7)	137 (50.0)		
Age										
<50 years old	118 (13.4)	1 (10.0)	51 (12.9)	66 (13.8)	0.82	0.85	83 (13.6)	35 (12.8)	7.57	0.06
50–60 years old	194 (22.0)	3 (30.0)	90 (22.7)	101 (21.2)			144 (23.7)	50 (18.2)		
60–70 years old	317 (35.9)	5 (50.0)	137 (34.6)	175 (36.7)			222 (36.5)	95 (34.5)		
>70 years old	254 (28.8)	1 (10.0)	118 (29.8)	135 (28.3)			159 (26.1)	95 (34.6)		
Local medical patient										
Yes	864 (97.8)	10 (100.0)	392 (99.0)	462 (96.9)	4.63	**0.03**	602 (99.0)	262 (95.3)	12.58	<**0.01**
No	19 (2.2)	0 (0.0)	4 (1.0)	15 (3.1)			6 (1.0)	13 (4.7)		
Employment status										
Retired	280 (31.7)	1 (10.0)	147 (37.1)^a^	132 (27.7)^a^	264.14	<**0.01**	198 (32.6)^*^	82 (29.8)^*^	25.76	<**0.01**
Formal employed	61 (6.9)	0 (0.0)	28 (7.1)^a^	33 (6.9)^a^			44 (7.2)	17 (6.2)		
Flexible employed	179 (20.3)	9 (90.0)	156 (39.4)^b^	14 (2.9)^b^			146 (24.0)^†^	33 (12.0)^†^		
Non-employed	363 (41.1)	0 (0.0)	65 (16.4)^c^	298 (62.5)^c^			220 (36.2)^‡^	143 (52.0)^‡^		
Total	883	10	396	477	—	—	608	275	—	—
			873							

Notes: Bold means that the distributions were statistically different (p < 0.05). Employment status subgroups with different superscripts implied statistically significant differences of patient distributions in 2017 and 2018; patient adopted gefitinib and icotinib for treatment after pairwise comparison with Bonferroni correction (p *<* 0.0083).

### Segmented Linear Regression Analysis of the Monthly Number of Patients Who Initiated Treatment With the Medicines of Interest Before and After Health Insurance Coverage

**FIGURE 1:**
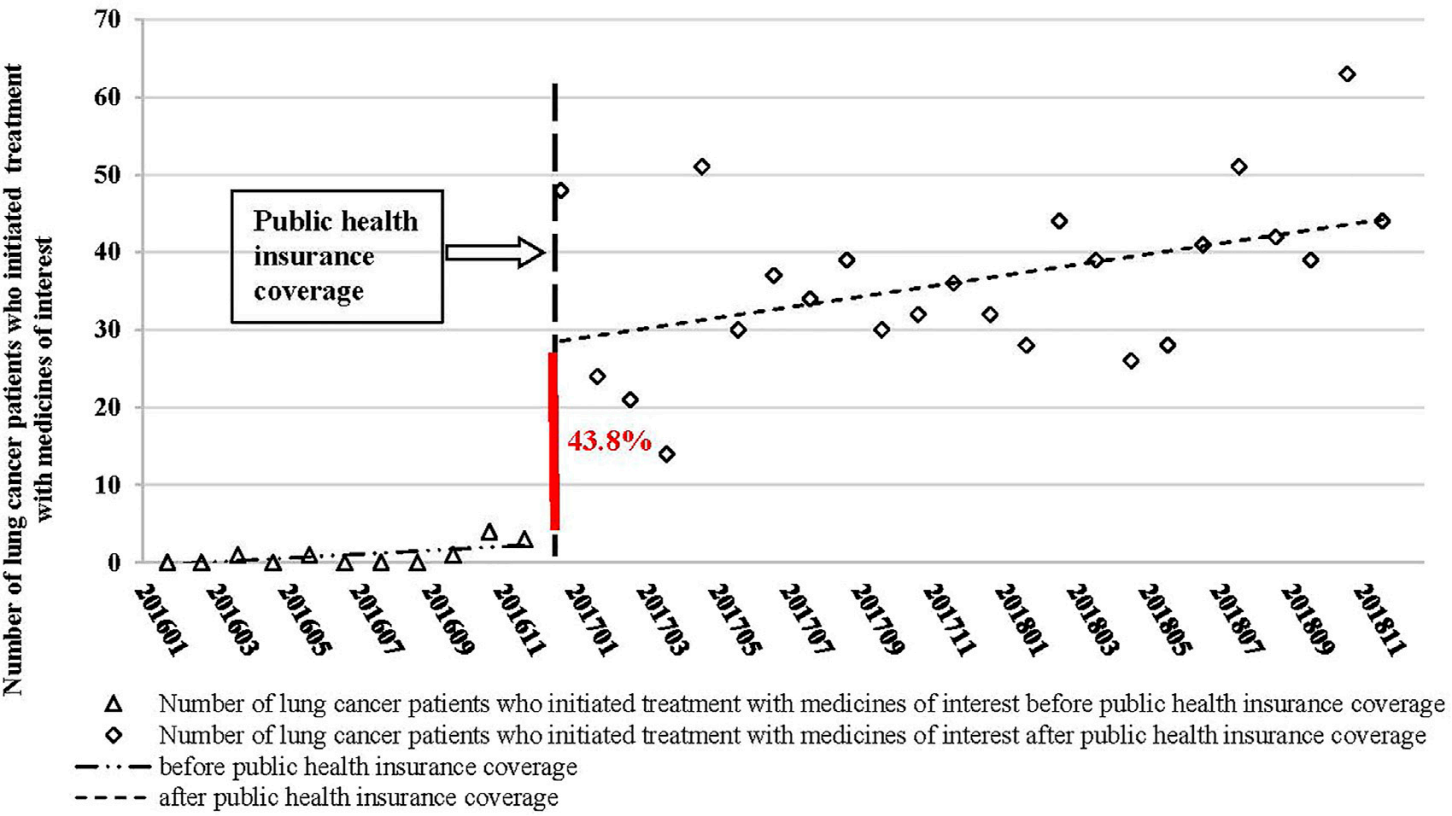
Segmented linear regression analysis results of the monthly number of patients who initiated treatment with the medicines of interest before and after the public health insurance coverage (January 2016–December 2018).

The segmented linear regression analysis showed that before health insurance coverage, the monthly number of patients who initiated treatment with the medicines of interest remained stable at a low level (p = 0.73). The number abruptly increased by 26 in January 2017, in the month that the health insurance coverage policy was implemented (95% CI: 14–37, p < 0.01). The monthly number remained increased significantly at a higher level after health insurance coverage (p < 0.01). The trend change was statistically significant (p < 0.01) (Figure 1 and Table 2).

**TABLE 2:** Segmented linear regression analysis results of the monthly number of patients who initiated treatment with medicines of interest before and after the public health insurance coverage.

Outcome variable	Coefficient		95% CI	p-value
Number of lung cancer patients who initiated treatment with medicines of interest	Intercept	−0.758	—	—
	Baseline trend	0.245	−1.168 to 1.657	0.726
	Level change	25.657	14.049–37.266	<0.001
	Trend change	0.438	0.159–1.936	<0.001
	Afterward trend	0.683	0.185–1.181	<0.001

### Multivariate Linear Regression Analysis of the Determinants of Patient Affordability of Lung Cancer Treatment With the Medicines of Interest

The goodness of fit of the fitted model R-squared was 0.50, and the Durbin–Watson test value was 1.94, which implied a non-significant auto-correlation of residuals. As presented in Table 3, by controlling the other factors, the proportionate OOP expenditures for lung cancer-related treatment with the medicines of interest of patients enrolled in the formal employee program not entitled to government-funded supplementary health insurance benefit and patients enrolled in the resident program were 18.3% (95% CI: 14.1–22.6, p < 0.001) and 26.7% (95% CI: 21.0–32.4, p < 0.001) higher than that of patients enrolled in the formal employee program with government-funded supplementary health insurance benefit. The proportionate OOP expenditure for lung cancer-related treatment with gefitinib was 10.4% higher than that of those treated with icotinib (95% CI: 7.9–2.9, p < 0.001).

**TABLE 3:** Multivariate linear regression analysis results of the proportionate OOP expenditure for lung cancer-related treatment with medicines of interest.

Variable	Coefficient	*SE*	95% CI		*t*	p-value
			Lower	Upper		
Age (ref.<50 years old)						
50–60 years old	0.028	0.022	−0.014	0.071	1.323	0.186
60–70 years old	0.013	0.022	−0.031	0.056	0.578	0.563
>70 years old	0.016	0.024	−0.031	0.063	0.679	0.498
Male (ref. Female)	−0.022	0.012	−0.045	0.002	−1.778	0.076
Local medical patient (ref. Non-local)	−0.002	0.041	−0.082	0.079	−0.037	0.970
Type of health insurance (ref. Formal employee program enrollee entitled to government-funded supplementary health insurance coverage)						
Formal employee program enrollee not entitled to government-funded supplementary health insurance coverage	**0.183**	0.022	**0.141**	**0.226**	8.506	<**0.001**
Resident program enrollee	**0.267**	0.029	**0.210**	**0.324**	9.211	<**0.001**
Status of employment (ref. formal employed)						
Retired	**-0.085**	0.027	**-0.137**	**-0.033**	-3.198	**0.001**
Flexible employed	-0.011	0.048	-0.106	0.083	-0.235	0.814
Medication (ref. Icotinib)	**0.104**	0.013	**0.079**	**0.129**	8.054	<**0.001**

Note: Bold implies statistically significant. Coefficient means the expected absolute changes of the proportionate OOP expenditure for lung cancer-related treatment with medicines of interest when the categorical variable shifted from the reference category to respective category, holding all the other variables constant, e.g., if the proportionate OOP of patients treated with icotinib was 30%, that of patients treated with gefitinib was 40.4%.

SE, standard error; CI, confidence interval; OOP, out of pocket.

## Discussion

The public health insurance coverage significantly increased the number of advanced or metastatic EGFR mutation-positive NSCLC patients under treatment with the medicines of interest. This finding was consistent with the results of other studies (Chen et al., 2018; Xian et al., 2019), especially in line with the results of a recent publication based on the hospital medical records in the same city during the same period, and targeted the novel anti-breast cancer medicines newly covered by the public health insurance program (Diao et al., 2021). Diao et al. found that the average proportion of human epidermal growth factor receptor 2 (HER2)-positive breast cancer patients who initiated medication with the medicines of interest increased from 37.4% before September 2017 to 69.2% afterward. In the month of public health insurance coverage, the number of HER2-positive breast cancer patients who initiated medication with the medicines of interest abruptly increased by 18.3% and continued to increase afterward. In general, public health insurance coverage of novel targeted anti-lung cancer medicines benefited the majority of patients. We estimated the medication cost of maintenance treatment for EGFR mutation-positive advanced or metastatic NSCLC based on the treatment guideline and the median progression-free survival time of 10 months reported by the published clinical studies (National Health Commission of the People’s Republic of China, 2019; Fujian Provincial People’s Government, 2017, 2018; Wang and Sun, 2020; Fuzhou Bureau of Statistics, 2019; Chinese Society of Clinical Oncology Guidelines Working Committee, 2018; Shi et al., 2013; Yang et al., 2017) and the local price and insurance reimbursement policies in 2018 (Fujian Provincial Department of Finance, 2018; Fuzhou Municipal People’s Government, 2018, 2019). The medication cost for 10-month maintenance treatment with iconitib to be paid by patient OOP was about US$4,300. Such a cost will be much higher and reach US$5,200 if treated with gefitinib. The low-income patient might tend to adopt cheaper medications, and the high-income patient might prefer more expensive newer therapies with potentially better clinical efficacy. Doctors always consulted the less well-off patients and those with poor benefits package patients for shifting to alternative therapies when there are cheaper products available (Yi-Ting et al., 2019; Trapani et al., 2019; Li and Li, 2018). This was also in line with the findings of this study that among patients with different employment statuses, the number and proportion of non-employed who initiated the maintenance targeted treatment with icotinib were significantly higher than those who initiated the maintenance targeted treatment with gefitinib. However, flexible employed patients with relatively high economic ability and less price sensitivity might be more likely to choose the newly included targeted medicines with potentially better efficacy. With an increasing number of novel medicines covered by the public health insurance program, the total number of patients under medications of interest will be an increasing trend overall, while the medications with different medicines of interest may vary due to the competition of interchangeable generics or bio-similars or the competition of alternative therapeutics with the same indication (Diao et al., 2019).

This study found that the determinant of the proportionate OOP expenditure for lung cancer-related treatment with the medicines of interest was the type of health insurance coverage of the patient. The proportionate OOP expenditures for lung cancer-related treatment with the medicines of interest of formal employee program enrollees without government-funded supplementary health insurance benefit and the resident program enrollees were significantly higher than that of those entitled to the supplementary benefit. Although public health insurance coverage of novel targeted anticancer medicines would reduce patient financial burden in general, some patients might still bear too heavy financial burden. The annual disposable per capita income of a Fuzhou rural resident in 2018 was US$4,850. An EGFR mutation-positive advanced or metastatic NSCLC rural resident patient had to spend 10–15 months of salary to pay the OOP expenditure of icotinib or gefitinib for the median progression-free survival time (10 months). Studies in other provinces drew similar conclusions that the economic burden of rural residents was much more serious than that of urban residents (Diao et al., 2019; Tian et al., 2017). Existing evidence in either China or many other countries also showed that higher OOP costs were associated with increased rates of abandonment of insurer-approved novel anticancer agents (Li et al., 2021; Wheeler et al., 2021; Doshi et al., 2018; Moye-Holz et al., 2018; Olszewski et al., 2017; Sruamsiri et al., 2015; Lammers et al., 2014; Schoen et al., 2009). High OOP costs and poor health insurance benefits packages were also found to be associated with poor financial wellbeing and highly likely reporting financial distress in Canada. High OOP expenses incurred by accessing cancer care showed direct associations with poor treatment adherence, decreased quality of life, and increased mortality (Pearse et al., 2020). Such consequences are not in line with the objective of the public health insurance coverage policy of novel anticancer medicines.

Assuming that all diagnoses and treatments complied with the national diagnosis and treatment guidelines in Fuzhou, the actual proportionate OOP expenditure for lung cancer-related treatment with the medicines of interest in the real clinical world should be determined by the choice of non-reimbursable care or therapy and benefits package of the patient. We did not find a significant difference in the utilization of non-reimbursable care or therapy between patients enrolled in different health insurance programs from their lung cancer-associated medical records. We would think that the health insurance benefits package might be the main driver of different proportionate OOP expenditures. Evidence about the restrictive insurance benefits and its negative implications on cancer patients in the United States showed that use of patient assistance program was partly because of restrictive local Medicaid policies (Felder et al., 2011) and strongly tied to insurance-imposed cost sharing (Olszewski et al., 2018). Not like the countries where there is a single payer for the whole population covered by the publicly funded health security program, the current public health insurance system in China consists of two parallel programs, the formal employee program and the resident program. It is jointly financed by the government, the society, and the individuals. Formal employees and their employers pay insurance contributions in different fixed proportions of payrolls. Residents pay lower contributions than the employees. The contributions of the residents are in different categories and linked with the ability to pay. Although subsidized by the government, the resident program has a lower funding level than the employee program and has been pooled and managed separately. Different program enrollees and residents with different abilities to pay are entitled to different annual deductibles, different proportions of insurance reimbursement, and different annual caps of insurance reimbursement. All patients have to pay totally OOP after the cap. Considering that there is a disparity of funding level between the formal employee program and the resident program and the benefits packages are linked with the ability to pay, the socioeconomically disadvantaged patients are very likely to encounter financial distress under such a system. Although the health financial aid program may further reduce the OOP of the patients in financial hardship, a significant number of patients may still be likely to fall into financial distress when their ability to pay is just above the threshold of the aid. This is especially the case for cancer patients enrolled in the resident program who need expensive treatment. Public health insurance coverage of novel targeted anticancer medicines based on such a system may benefit the high-income patient but may not be accessible at all for the low-income patients (Si et al., 2017). Only if the benefits package of the low-income patients is further strengthened could they benefit from the public health insurance coverage of novel targeted anticancer medicines. To achieve the policy goal of not to leave anyone behind, there is an urgent need to reform the public health insurance system. As recommended by Olszewski et al. (2018), shifting from the percentage-based coinsurance to fixed copayments may ensure a more predictable OOP burden. Defining an appropriate basic benefits package for all regardless of the ability to pay, revoking the insurance reimbursement cap, and building a safety net for the patient who encounters catastrophic expenditure are all possible approaches to help achieve the policy goal of not to leave anyone behind.

In conclusion, the public health insurance coverage of novel targeted anti-lung cancer medicines benefited the patient generally. However, patients with low ability to pay and weak basic health insurance benefits package had a much higher proportionate OOP expenditure for treatment with the newly covered novel medications than the high-income patient. To enable that patient benefit from the public health insurance coverage of novel targeted anticancer medicines more equally and thoroughly, and not to leave anyone behind, it is necessary to strengthen the benefits package of the resident program, and to optimize the current financing mechanism of the public health insurance system.

To the best of our knowledge, this is the first study to analyze who and how cancer patients benefited from the public health insurance coverage of novel targeted anticancer medicines in the real world based on the health insurance claim data. The study was based on several assumptions. Firstly, the diagnoses and treatments of the studied patients all followed the national guidelines and regulations and the health insurance covered medications with the studied medicines followed the indication requirements of the basic health insurance programs. Secondly, our review of the local health-related policies showed that no other critical policies related to the use of novel targeted anticancer medicines and cancer treatment were introduced in Fujian province during the observation period. In addition, the epidemiological characteristics of the insured patients and the diagnosis and treatment behaviors of doctors and patients did not change significantly during the study period. Besides, hospital pharmacies were the primary source of medicines to patients in Fuzhou. This study was conducted based on the health insurance claim data, which did not consider the situation of purchasing medicines outside the hospital or through the Patient Assistance Program (PAP) and might underestimate the medication expenditure. However, as China already achieved universal health insurance coverage and expensive anticancer medicines dispensed from retail pharmacies were not reimbursed by the public health insurance program in Fuzhou city, purchases outside hospitals could be neglected. There was no PAP in Fuzhou city for icotinib. For gefitinib, considering that the precondition of access to the PAP was to complete at least 8 months of paid treatment and it only targeted the patients who were entitled to the subsistence allowance (who were only less than 0.1% of the total population of Fuzhou city) (Fuzhou Bureau of Statistics, 2019), the number of treatments through PAP could also be neglected. Moreover, the study did not include the analysis of the income of the studied patients and indirect medical expenditures. The proportionate OOP expenditure can only reflect patient affordability from one aspect. There is a need for future studies to collect income information and calculate the indirect medical expenses of the patient to conduct a comprehensive analysis of the medical economic burden of the patient. In addition, females included in this study is a bit higher than the males, while lung cancer affects more males than females in general. This is because the included patients for this study were the advanced or metastatic EGFR mutation-positive NSCLC patients enrolled in the public health insurance in Fuzhou city who used the expensive novel anti-lung cancer medicine newly covered by the insurance. There are many factors affecting the progression of EGFR mutation-positive NSCLC patients to advanced or metastatic and the survival of these patients to approach expensive treatment. Such factors might affect the male and the female differently. Further studies are needed to identify the in-depth reasons. Apart from this, Fujian province has been a pioneer province in terms of reforming the health insurance system and the management of medications. The results of this study might not reflect the implementation of the health insurance coverage policy of novel targeted anticancer medicines in other areas. There is a need to include more regions in future studies to gain a comprehensive mapping of the policy implementation across the country.

## Data Availability

The raw data supporting the conclusion of this article will be made available upon request with appropriate reason by the authors without undue reservation.

## References

[B1] Allemani C. Matsuda T. Di Carlo V. Harewood R. Matz M. Nikšić M. (2018). Global Surveillance of Trends in Cancer Survival 2000-14 (CONCORD-3): Analysis of Individual Records for 37 513 025 Patients Diagnosed with One of 18 Cancers from 322 Population-Based Registries in 71 Countries. Lancet 391, 1023–1075. 10.1016/S0140-6736(17)33326-3 29395269 PMC5879496

[B2] Central People's Government of the People's Republic of China (2020). Opinions of the CPC Central Committee and the State Council on Deepening the Reform of the Medical Security System. Retrieved from: http://www.gov.cn/zhengce/2020-03/05/content_5487407.htm (accessed April 16, 2021).

[B3] Chen W. Zheng R. Baade P. D. Zhang S. Zeng H. Bray F. (2016). Cancer Statistics in China, 2015. CA Cancer J. Clin. 66, 115–132. 10.3322/caac.21338 26808342

[B4] Chen W. Q. Zheng R. S. Zeng H. M. Zhang S. W. Zhao P. He J. (2012). [Trend Analysis and Projection of Cancer Incidence in China between 1989 and 2008]. Zhonghua Zhong Liu Za Zhi 34, 517–524. 10.3760/cma.j.issn.0253-3766.2012.07.010 22967471

[B5] Chen Z. Leng J. Gao G. Liu Y. (2018). Anticancer Agents Incorporated into Medical Insurance Policy: Taking a Tertiary Oncology Institution in Beijing as an Example. Chin. Health Econ. 37, 30–34. 10.1016/S0140-6736(18)32666-7

[B6] Chinese Society of Clinical Oncology Guidelines Working Committee (2018). Chinese Society of Clinical Oncology (CSCO) Guidelines for the Diagnosis and Treatment of Primary Lung Cancer. Beijing, China: People's Medical Publishing House.

[B7] Chou Y.-T. Farley J. F. Stinchcombe T. E. Proctor A. E. Lafata J. E. Dusetzina S. B. (2019). The Association between Medicare Low-Income Subsidy and Anticancer Treatment Uptake in Advanced Lung Cancer. J. Natl. Cancer Inst. 112, 637–646. 10.1093/jnci/djz183 PMC7301149 31501872

[B8] Diao Y. Lin M. Xu K. Huang J. Wu X. Li M. (2021). How Government Health Insurance Coverage of Novel Anti-cancer Medicines Benefited Patients in China - a Retrospective Analysis of Hospital Clinical Data. BMC Health Serv. Res. 2121 (1), 856. 10.1186/s12913-021-06840-3 PMC8380313 34419013

[B9] Diao Y. Qian J. Liu Y. Zhou Y. Wang Y. Ma H. (2019). How Government Insurance Coverage Changed the Utilization and Affordability of Expensive Targeted Anti-cancer Medicines in China: an Interrupted Time-Series Study. J. Glob. Health 9, 020702. 10.7189/jogh.09.02060310.7189/jogh.09.020702 31673344 PMC6815654

[B10] Doshi J. A. Li P. Huo H. Pettit A. R. Armstrong K. A. (2018). Association of Patient Out-Of-Pocket Costs with Prescription Abandonment and Delay in Fills of Novel Oral Anticancer Agents. J. Clin. Oncol. 36 (5), 482. 10.1200/JCO.2017.74.5091 29261440

[B11] Economic Information Daily (2018). Provincial Pooled Procurement of Anti0cancer Medicines in China. Retrieved from: http://www.xinhuanet.com/fortune/2018-07/12/c_1123113325.htm (accessed April 16, 2021).

[B12] Ezeife A. D. Morganstein B. J. Lau S. Law J. H. Le L. W. Bredle J. (2018). Financial burden Among Patients with Lung Cancer in a Publically Funded Health Care System - Sciencedirect. Clinical Lung Cancer, 20(4), 231–236. 10.1016/j.cllc.2018.12.010 30797721

[B13] Fang P. Zhao S. Li Q. Zhang X. (2017). Discussing the Management Strategy of Medical Insurance of High Price Drugs in China. Chin. Health Serv. Manage. 11, 826–828. CNKI:SUN:ZWSG.0.2017-11-010

[B14] Felder T. M. Lal L. S. Bennett C. L. Franzini L. (2011). Cancer Patients’ Use of Pharmaceutical Patient Assistance Programs in the Outpatient Pharmacy at a Large Tertiary Cancer center. Community Oncol. 8, 279–286. 10.1016/S1548-5315(12)70023-2 22879815 PMC3413301

[B15] Fujian Provincial Department of Finance (2018). Notice of the Fujian Provincial Medical Security Management Committee Office on Adjusting the Reimbursement Level for the National Negotiated Medicines. Retrieved from: http://czt.fujian.gov.cn/zfxxgkzl/zfxxgkml/qt/qt/201812/t20181217_4710614.htm (accessed April 16, 2021).

[B16] Fujian Provincial People's Government (2017). Notice on Implementing the Ministry of Human Resources and Social Security to Include 36 Kinds of Drugs in Category B of the National Basic Medical Insurance, Work-Related Injury Insurance and Maternity Insurance Catalogues. Retrieved from: http://www.fujian.gov.cn/zc/zxwj/bmwj/201709/t20170926_1311983.htm (accessed April 16, 2021).

[B17] Fuzhou Bureau of Statistics (2019). Fuzhou Statistical Yearbook 2019. Beijing, China: China Statistics Press. Retrieved from: http://tjj.fuzhou.gov.cn/zz/fztjnj/2019tjnj/indexch.htm (accessed April 16, 2021).

[B18] Fuzhou Municipal People's Government (2018). Guide to Basic Medical Insurance for Employees in Fuzhou. Retrieved from: http://mqx.fuzhou.gov.cn/xjwz/zwgk/zfxxgkzdgz/yl/ybxx/201809/t20180921_2611201.htm (accessed April 16, 2021).

[B19] Fuzhou Municipal People's Government (2019). Guide to Rural and Urban Residents Basic Medical Insurance in Fuzhou. Retrieved from: http://www.fuzhou.gov.cn/zgfzzt/sybzx/ylbzjj/zchb/201912/t20191213_3129106.htm . (accessed 20 March 2021).

[B20] Health News (2018). Policy Linkage to Reduce the burden of Cancer Patients' Drug Costs. Retrieved from: http://www.nhc.gov.cn/yaozs/s3578m/201805/266e0ff7b3ee4ff3adebed6b110023b7.shtml (accessed April 16, 2021).

[B21] IQVIA Institute (2018). Global Oncology Trends 2018. Innovation, Expansion and Disruption. Retrieved from: https://www.iqvia.com/institute/reports/global-oncology-trends-2018 (accessed April 16, 2021).

[B22] Lammers P. Criscitiello C. Curigliano G. Jacobs I. (2014). Barriers to the Use of Trastuzumab for HER2+ Breast Cancer and the Potential Impact of Biosimilars: A Physician Survey in the United States and Emerging Markets. Pharmaceuticals (Basel) 7 (9), 943–953. 10.3390/ph7090943 PMC4190498 25232798

[B23] Li H. Liu S. (2018). Molecular Targeted Therapy for Non-small Cell Lung Cancer: the Reality in China and Coping Strategy. Pract. J. Med. Pharm. 4, 373–375+379. 10.14172/j.issn1671-4008.2018.04.030

[B24] Li M. Diao Y. Ye J. Sun J. Jiang Y. (2021). Analysis of How Public Health Insurance Coverage of Targeted Anti-breast Cancer Medicines through National Medicines Price Negotiation Benefited Patient. J. Pharmaco-epidemiolog 30 (6), 53–59. CNKI:SUN:YWLX.0.2021-06-008

[B25] Ma J. Jemal A. Fedewa S. A. Islami F. Lichtenfeld J. L. Wender R. C. (2019). The American Cancer Society 2035 challenge Goal on Cancer Mortality Reduction. CA Cancer J. Clin. 69, 351–362. 10.3322/caac.21564 31066919

[B26] Ministry of Human Resources and Social Security (2017). Notice Regarding the Inclusion of 36 Types of Drugs in the Scope of Class B of the National Basic Medical Insurance, Work Injury Insurance and Maternity Insurance Drug Catalogue. Retrieved from: http://www.mohrss.gov.cn/gkml/zlbmxgwj/ylbx_3063/201707/t20170718_274153.html (accessed April 16, 2021).

[B27] Moye-Holz D. Soria Saucedo R. van Dijk J. P. Reijneveld S. A. Hogerzeil H. V. (2018). Access to Innovative Cancer Medicines in a Middle-Income Country - the Case of Mexico. J. Pharm. Pol. Pract 11 (25), 25. 10.1186/s40545-018-0153-y PMC6199792 30386627

[B28] National Health and Family Planning Commission (2016). Notice of the General Office of the National Health and Family Planning Commission on Announcing the Results of National Drug Price Negotiations. Retrieved from: http://www.nhfpc.gov.cn/yaozs/s7655/201605/58c5bc1ed0f14c75b8f15f1c149b35f4.shtml (accessed April 16, 2021).

[B29] National Health Commission of the People’s Republic of China (2019). Primary Lung Cancer Diagnosis and Treatment Specification (2018 Edition). J Multidiscip Cancer Manage 5, 120. 10.12151/JMCM.2019.03-16

[B30] National Healthcare Security Administration (2018). Notice of the National Medical Security Administration on the Inclusion of 17 Anticancer Drugs in the National Basic Medical Insurance, Work Injury Insurance, and Maternity Insurance Drug Catalogues in Category B. Retrieved from: http://www.nhsa.gov.cn/art/2018/10/10/art_19_397.html (accessed April 16, 2021).

[B31] National Healthcare Security Administration. (2019). Notice on the Inclusion of Negotiation Medicines in the Category B List of the National Basic Medical Insurance, Professional Injury Insurance and Maternity Insurance. Retrieved from: http://www.nhsa.gov.cn/art/2019/11/28/art_37_2050.html . (accessed 20 March 2021).

[B32] Olszewski A. J. Dusetzina S. B. Eaton C. B. Davidoff A. J. Trivedi A. N. (20172017). Subsidies for Oral Chemotherapy and Use of Immunomodulatory Drugs Among Medicare Beneficiaries with Myeloma. J. Clin. Oncol. 35 (29), 3306–3314. 10.1200/JCO.2017.72.2447 PMC5652870 28541791

[B33] Olszewski A. J. Zullo A. R. Nering C. R. Huynh J. P. (2018). Use of Charity Financial Assistance for Novel Oral Anticancer Agents. J. Oncol. Pract. 14 (4), e221. 10.1200/JOP.2017.027896 29443649 PMC5951296

[B34] Pearse W. B. Altman J. K. Kircher S. M. (2020). Perceptions of Financial and Psychosocial burden Among Patients with Hematologic Malignancies Treated with Novel Oral Antineoplastic Agents. Blood 136 (Suppl. 1), 20. 10.1182/blood-2020-141383

[B35] Schoen C. Stremikis K. Collins S. Davis K. (2009). Progressive or Regressive? A Second Look at the Tax Exemption for Employer-Sponsored Health Insurance Premiums. Issue Brief (Commonw Fund) 53, 1–8. 19449499

[B36] Shi Y. Sun Y. Ding C. Wang Z. Wang C. Bai C. (2016). [China Experts Consensus on Icotinib for Non-small Cell Lung Cancer Treatment (2016 version)]. Zhongguo Fei Ai Za Zhi 19, 489–494. 10.3779/j.issn.1009-3419.2016.07.12 27339727 PMC5972963

[B37] Shi Y. Zhang L. Liu X. Zhou C. Zhang L. Zhang S. (2013). Icotinib versus Gefitinib in Previously Treated Advanced Non-small-cell Lung Cancer (ICOGEN): a Randomised, Double-Blind Phase 3 Non-inferiority Trial. Lancet Oncol. 14 (10), 953–961. 10.1016/S1470-2045(13)70355-3 23948351

[B38] Si L. Chen M. Palmer A. J. (2017). Has Equity in Government Subsidy on Healthcare Improved in China? Evidence from the China's National Health Services Survey. Int. J. Equity Health 16, 6. 10.1186/s12939-017-0516-z 28069001 PMC5223563

[B39] Siegel R. L. Miller K. D. Jemal A. (2020). Cancer Statistics, 2020. CA Cancer J. Clin. 70, 7–30. 10.3322/caac.21590 31912902

[B40] Sruamsiri R. Ross-Degnan D. Lu C. Y. Chaiyakunapruk N. Wagner A. K. (2015). Policies and Programs to Facilitate Access to Targeted Cancer Therapies in Thailand. PLOS One 10 (3), e0119945. 10.1371/journal.pone.0119945 25798948 PMC4370712

[B41] Tian M. Cui D. Zhang Y. Yin X. Fang X. Hu J. (2017). Affordability Evaluation for 3 Kinds of Anti-tumor Targeted Drugs: Taking Hubei Province as an Example. Chin. Pharm. 28 (20), 2746–2749. 10.6039/j.issn.1001-0408.2017.20.03

[B42] Trapani D. Curigliano G. Eniu A. (2019). Breast Cancer: Reimbursement Policies and Adoption of New Therapeutic Agents by National Health Systems. Breast Care (Basel) 14, 373–381. 10.1159/000502637 31933583 PMC6940432

[B43] Wang L. (2016). The Exploration of Medical Insurance in Precise Poverty Alleviation in Inner Mongolia---Bringing Tumor Targeting Drugs into the Payment of Medical Insurance. Chin. Health Insur 3, 48–50. 10.369/j.issn.1674-3830.2016.3.009

[B44] Wang W. Sun Q. (2020). Analysis and Countermeasures of the Employment Status of Flexible Workers. Econ. Res. Guide 2, 68–69. CNKI:SUN:JJYD.0.2020-02-024

[B45] Wheeler S. B. Spees L. P. Jackson B. E. Baggett C. D. Dinan M. A. (2021). Patterns and Predictors of Oral Anticancer Agent Use in Diverse Patients with Metastatic Renal Cell Carcinoma. JCO Oncol. Pract. 20, 01082. 10.1200/OP.20.01082 PMC8678030 34138665

[B46] Xian Y. Xue Z. Gu J. (2019). The Reform and Effect of Anti-cancer Targeted Medicines Management System in a Tertiary General Hospital. Med. J. Commun. 33, 655–657+660. 10.19767/j.cnki.32-1412.2019.06.042

[B47] Yang J. J. Zhou C. Huang Y. Feng J. Lu S. Song Y. (2017). Icotinib versus Whole-Brain Irradiation in Patients with EGFR-Mutant Non-small-cell Lung Cancer and Multiple Brain Metastases (BRAIN): a Multicentre, Phase 3, Open-Label, Parallel, Randomised Controlled Trial. Lancet Respir. Med. 5 (9), 707–716. 10.1016/S2213-2600(17)30262-X 28734822

[B48] Zeng H. Chen W. Zheng R. Zhang S. Ji J. S. Zou X. (2018). Changing Cancer Survival in China during 2003-15: a Pooled Analysis of 17 Population-Based Cancer Registries. Lancet Glob. Health 6, e555. 10.1016/S2214-109X(18)30127-X 29653628

[B49] Zeng H. M. Zheng R. S. Zhang S. W. Zhao P. He J. Chen W. Q. (2012). [Trend Analysis of Cancer Mortality in China between 1989 and 2008]. Zhonghua Zhong Liu Za Zhi 34, 525–531. 10.3760/cma.j.issn.0253-3766.2012.07.011 22967472

[B50] Zheng R. S. Sun K. X. Zhang S. W. Zeng H. M. Zou X. N. Chen R. (2019). [Report of Cancer Epidemiology in China, 2015]. Zhonghua Zhong Liu Za Zhi 41, 19–28. 10.3760/cma.j.issn.0253-3766.2019.01.00810.3760/cma.j.issn.0253-3766.2019.01.005 30678413

